# Verbal and facial-emotional Stroop tasks reveal specific attentional interferences in sad mood

**DOI:** 10.1002/brb3.38

**Published:** 2012-01

**Authors:** Linda Isaac, Janna N Vrijsen, Paul Eling, Iris van Oostrom, Anne Speckens, Eni S Becker

**Affiliations:** 1 Clinical Psychology and Behavioural Science Institute, Radboud University NijmegenNijmegen, The Netherlands; 2 Department of Psychiatry, Radboud University Nijmegen Medical CentreNijmegen, The Netherlands; 3 Donders Institute for Brain, Cognition and Behaviour, Radboud UniversityNijmegen, The Netherlands

**Keywords:** Cognitive bias, face processing, mood, Stroop

## Abstract

Mood congruence refers to the tendency of individuals to attend to information more readily when it has the same emotional content as their current mood state. The aim of the present study was to ascertain whether attentional interference occurred for participants in sad mood states for emotionally relevant stimuli (mood-congruence), and to determine whether this interference occurred for both valenced words and valenced faces. A mood induction procedure was administered to 116 undergraduate females divided into two equal groups for the sad and happy mood condition. This study employed three versions of the Stroop task: color, verbal-emotional, and a facial-emotional Stroop. The two mood groups did not differ on the color Stroop. Significant group differences were found on the verbal-emotional Stroop for sad words with longer latencies for sad-induced participants. Main findings for the facial-emotional Stroop were that sad mood is associated with attentional interference for angry-threatening faces as well as longer latencies for neutral faces. Group differences were not found for positive stimuli. These findings confirm that sad mood is associated with attentional interference for mood-congruent stimuli in the verbal domain (sad words), but this mood-congruent effect does not necessarily apply to the visual domain (sad faces). Attentional interference for neutral faces suggests sad mood participants did not necessarily see valence-free faces. Attentional interference for threatening stimuli is often associated with anxiety; however, the current results show that threat is not an attentional interference observed exclusively in states of anxiety but also in sad mood.

## Introduction

A central interest in the study of mood is the interplay between mood and cognition. In this particular domain, the mood-congruency hypothesis is of key relevance. According to this hypothesis, positive mood should facilitate information processing of positive information and negative mood should facilitate information processing of negative information (Bower 1981). Mood induction methods help us to gain insights into the question of how mood affects cognitive processes in a systematic way and therefore have become a widely used technique to investigate the interplay between mood and cognition (for a review, see Gotlib and Joormann 2010). There is ample evidence that people in a happy mood show selective attention for positive stimuli (Rowe et al. 2007). Accumulating evidence demonstrates that sad mood is associated with an attentional bias that functions in favor of processing emotionally negative information. Such attentional biases have been observed in emotional disturbances such as sad mood (Beck et al. 1987; Gilboa–Schechtman et al. 2000; Niedenthal et al. 2000), clinical depression (Gotlib et al. 2004), and anxiety (Rapee and Heimberg 1997; Koster et al. 2006). Some studies regarding cognitive processes in sad mood point to an attentional bias for negative content (e.g., McCabe et al. 2000), but others have failed to find such an attentional bias (MacLeod et al. 1986; Mogg et al. 1993). There is little corresponding literature on facial emotion perception and sad mood in normal participants. Bouhuys et al. (1995) reported a study in which facial emotion recognition was compared in normal healthy adults after sad and happy mood inductions and no effect of sad mood was observed. Recently, Chepenik et al. (2007) showed that sad mood affected memory for both emotional words and facial emotion recognition in a healthy sample experimentally put into a sad mood state.

It is reasonable to speculate that this divergence in the literature is attributable to variations in methodology. For instance, it has been conjectured that unlike emotional words, the processing of valenced pictures is rooted in the semantic system and has “privileged access” to networks involved in both processing and storing affective information (Bradley et al. 1997). Evidence for this supposition has been put forth by De Houwer and Hermans (1994), who confirmed that while valenced pictures interfered with the categorization of valenced words, valenced word distracters failed to interfere with valenced pictorial categorization.

Considering the inherent information emotional faces convey about interpersonal evaluation, a topic that is of high relevance to the study of the effects of sad mood states (Davidson et al. 1989), it follows that we, along with others (e.g., Langenecker et al. 2005; Joormann and Gotlib 2007) argue that studying emotional faces is critical to understanding sad mood. The basic Stroop technique involves naming the color of the ink the word is written in and ignoring the meaning of the word text (Stroop 1935) and has been extended to examine information processing of emotional content. A number of studies have compared emotionally impacted and emotionally intact participants with regards to the time taken to name colors of negative words compared to neutral and positive items. The interpretations of both the color Stroop and the emotional Stroop tests imply the suppression of responses to distracting word information. In the work of Gotlib and McCann (1984), the emotional variant of the Stroop task illustrated that clinically depressed participants were slower to name the color of depressing words compared to nondepressing words due to difficulty inhibiting rumination triggered by negative words. This finding was replicated in a sample of sad-induced participants (Gilboa-Schechtman et al. 2000). It is noteworthy to mention the Stroop paradigm is limited insofar as attention is conceptualized as a single process, when in fact attentional processes include both engagement (excitation) and disengagement (inhibition), that are not easily disentangled by the Stroop task (Kahneman and Treisman 1984). Nonetheless, it continues to be a useful tool in examining attentional interference for mood-relevant content. Once again, with respect to mood research, some studies have found mood-congruency effects whereby individuals in a sad mood take longer to attend to depressive stimuli compared to happy mood individuals (Bower and Forgas 2001), whereas others have not found this bias (Bouhuys et al. 1997) in sad mood. Specifically, Stroop interference has been observed for sad words after sad mood induction in one study (Gilboa-Schechtman et al. 2000), but not in another (Perez et al. 1999). According to Chepenik et al. (2007), the literature contains relatively few studies on the impact of sad mood on cognitive processes other than memory with reported sad mood effects on facial emotion recognition and attention being relatively scarce. Although most recently research has shown mood-congruent effects for facial expressions in sad mood (Schmid and Schmid-Mast 2010).

The main purpose of the present study was to examine attentional interference among participants in a sad mood state by determining interference for mood-congruent stimuli (e.g., sad faces) and to establish whether this interference has a common mechanism influencing both emotional words and emotional faces. This research sought to examine both emotional words and emotional faces across four principal emotions to address as closely as possible, what captures the attention of people in a sad mood compared to those in a happy mood. Bearing this in mind, we specifically intended to evaluate attentional interference for the most socially salient of pictorial images: emotional faces. The inclusion of both sad and angry facial emotions will allow us to investigate if sad-induced participants have a mood-congruent bias for sad faces alone or a bias for negative faces in general (sad and angry faces). We hypothesized that:

1. Participants induced into a sad mood will show greater attentional interference for mood-congruent stimuli and this interference will be observed for both emotional words and faces, measured by longer response latencies for both depressive words in the verbal domain and sad faces in the visual domain.
2. Happy mood participants will demonstrate mood-congruence and cross-modality indexed by longer response latencies for positive stimuli (positive words/happy faces).

## Method

### Participants

One-hundred and twenty-four undergraduate females signed up for the experiment in exchange for study credit. Eight participants were excluded for nonfluency in Dutch, leaving the final sample of 116 female university students with a mean age of 20.9 (SD = 2.9) years. Participation was restricted to females to control for potential gender influences and Dutch fluency to control for potential confounds on the verbal-emotional Stroop task. All study participants were further screened for current and past mood complaints, cognitive impairments, color vision, and dyslexia.

### Materials

#### Questionnaires

All participants completed the Dutch versions of the State-Trait Anxiety Inventory (STAI) (Van der ploeg et al. 1980), Positive Affect and Negative Affect Scale (Peeters et al. 1996), and finally the Beck Depression Inventory (BDI) (Bouman et al. 1985).

#### Mood induction films and mood rating scale

Mood induction movie clips consisted of Happy Feet for the positive mood and Sophie's Choice for the sad mood. These specific film segments have been validated and proven to be reliable in previous studies (Fitzgerald et al. 2011) and in general, movie segments are considered a highly reliable technique for inducing mood (Westermann et al. 1996). Both the sad and happy mood induction consisted of a 12-min clip and a 7-min clip given at two separate time intervals. Participants were instructed to identify with the protagonist in the movie and “get into the same mood.” Mood ratings were collected using a computerized visual analogue scale that ranged from −10 (indicating saddest mood) to 10 (indicating happiest mood).

#### Color and verbal-emotional Stroop

A modified computerized Stroop color-naming task with emotional as well as color words was used. The Stroop consisted of five blocks of three trials each: sad words, happy words, fearful words, neutral words, and color words. A practice trial, containing 15 words selected from the different blocks, preceded the testing phase. The valenced words (from a Dutch translation of the Affective Norms for English Words database, Bradley and Lang 1999) were matched for length, frequency, and valence strength (see Table 1). The color trials contained the words “red,”“yellow,”“green,” and “blue.” The color block was always presented last, while the order of the other blocks was randomized across participants. The trials contained 48 words each and were sorted in four different columns. Within a block, the words were presented in a different order. Each of the eight selected words was presented five times per in a random order. The order of the colors was random as well. However, a restriction was set that the same word or ink color could not occur more than twice in a row. All stimuli appeared in lowercase Arial font (regular) and in font size 27. The projected stimuli appeared on the computer screen as color words presented against a black background.

**Table 1:** Stimuli from the verbal-emotional Stroop task.

Happy	Neutral	Sad	Fearful	Color
voldoening (satisfaction)	zegel (Seal)	zorgelijk (worrying)	alarm (alarm)	blauw (blue)
vrijheid (freedom)	onderdeel (part)	waardeloos (worthless)	moordenaar (killer)	geel (yellow)
sociaal (social)	gebeurtenis (event)	ontmoedigd (discouraged)	gewelddadig (violent)	rood (red)
interessant (interesting)	aanduiding (indicator)	schuldig (guilty)	bedreigend (threatening)	groen (green)
Heerlijk (delicious)	deurknop (doorknob)	verlies (loss)	gevaar (danger)	
gelukkig (happy)	geurloos (odorless)	verdriet (grief)	stikken (suffocate)	
vriendschap (friendship)	programma (program)	zinloos (senseless)	doodsangst (agony)	
zonnig (sunny)	instructie (instruction)	ongelukkig (unhappy)	bloedend (bleeding)	

#### Facial-emotional Stroop

**Figure 1:**
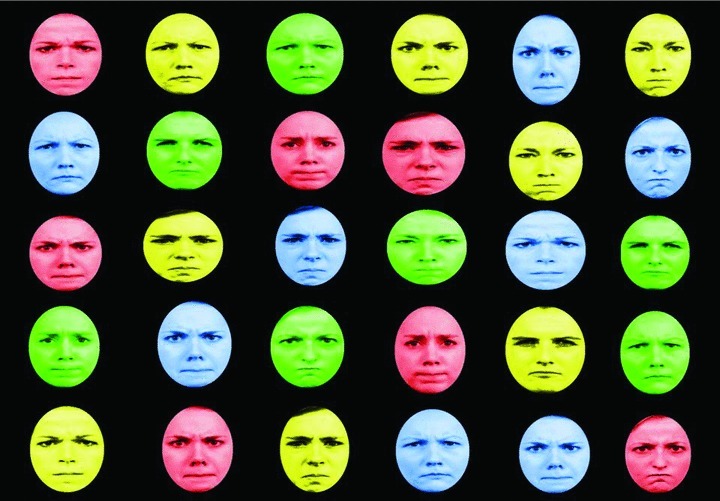
Sample angry female stimuli taken from the facial-emotional Stroop task.

**Figure 2:**
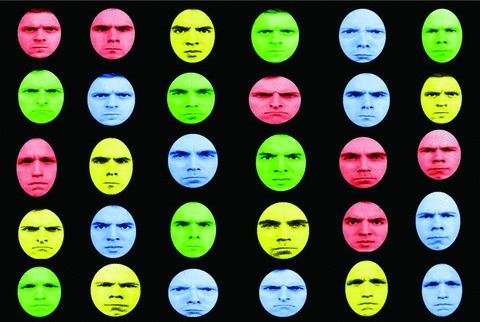
Sample angry male stimuli taken from the facial-emotional Stroop task.

A modified computerized Stroop color-naming task with emotional faces was used. Colored chairs were also included to offer a baseline measure of visual processing for complex objects. The facial-emotional Stroop consisted of eight blocks of three trials each: depressed-female faces, depressed-male faces, angry-male faces, angry-female faces, neutral-female faces, neutral-male faces, happy-female faces, happy-male faces. Colored chairs were also presented across the four Stroop colors as a control condition. A practice session containing five faces and five chairs, selected from the different blocks, preceded the testing phase. A total of 576 emotional faces were created for the facial-emotional Stroop from 36 different identities (18 males/18 females) × 4 emotions (happy, sad, neutral, and angry) × 4 Stroop colors (yellow, green, blue, and red) × 2 genders (male/female). Valenced faces were selected from the validated Radboud Faces database (Langner et al. 2010). A direct resemblance to the conventional Stroop was created by applying a color filter over each face and chair using the GNU Image Manipulation Program for Windows systems (GIMP, 2.6) matched on dimensions of color hue, saturation, contrast, and dimensions (960 × 720). Faces were cropped, free from hair or other external attributes that could serve as distracters or distinguishers. The order of all blocks was randomized per participant. The Stroop trials contained 30 faces each and were sorted in five different columns. Within a block, the faces were presented in a different order during every trial. The order of the colors was also random. A restriction was set that the same face could not occur more than twice in a row. The projected stimuli appeared on the computer screen as color faces were presented against a black background. See Figures 1 and 2.

### Procedure

Participants were randomly allocated to the sad or happy mood induction condition. Upon arrival in the laboratory, they were seated at a desk in an experimental cubicle, 50 cm away from the computer monitor. Prior to experimental testing, all participants first provided their consent for participation followed by a general screening questionnaire and the completion of all questionnaires. The experimental task was presented on a PC with a 17-inch (43-cm) color monitor (Dell Trinitron, Texas, USA). Participants sat in a comfortable chair facing the monitor at a distance of 70 cm. In an order-counterbalanced fashion, participants received either the verbal-emotional Stroop or the facial-emotional Stroop first following mood induction. The conventional Stroop always followed the verbal-emotional Stroop. The order of Stroop tasks and recording of response latencies was managed via Inquisit software (Millisecond Software, 2001, Version 1.33). Prior to viewing the first mood induction film, each participant was asked to rate their current mood from −10 to 10 on the mood rating scale described above. This provided the baseline mood rating. Thereafter, they were instructed to watch and listen to the film by placing the headphones on for film auditory and noise distraction control. They viewed either the sad or happy 12-min movie clip and were explicitly instructed to identify with the protagonist in the film. After viewing the first movie clip, participants were presented with the mood rating scale for the second time (postmood induction rating 1). Then they proceeded to the first Stroop task. Participants were instructed to name out loud the ink color (red, yellow, green, blue) and to indicate when the last ink color of that sheet was named by saying “done.” They worked along the top row from left to right and subsequently, without pausing, along each succeeding row. After each Stroop trial, the experimenter pressed the spacebar immediately to register the reaction time and then the next Stroop trial appeared. The experimenter was blind to all task conditions seated in the opposite direction of the computer screen. Following the first Stroop task, participants were instructed to watch and listen to the 7-min mood induction movie clip. Following this second mood induction, participants were asked to complete the mood rating scale for the third and final time (postmood induction rating 2). The remaining Stroop tasks were completed with the exact instructions as the first Stroop.

## Results

### Group characteristics and questionnaire measures

The means of the two mood groups were compared via independent group *t*-tests on the BDI, the Positive Affect Negative Affect Schedule (PANAS positive and negative scores), and the STAI (both trait and state scores). The results of the *t*-tests indicate the two mood groups did not differ significantly in their mean levels of depression (*t*(114) = 0.310, *P*= 0.757), positive affect (*t*(114) = 1.102, *P*= 0.273), negative affect (*t*(114) = 0.441, *P*= 0.660), state anxiety (*t*(114) = 1.049, *P*= 0.297), or trait anxiety (*t*(114) = 0.629, *P*= 0.531).

### Experimental mood induction

**Figure 3:**
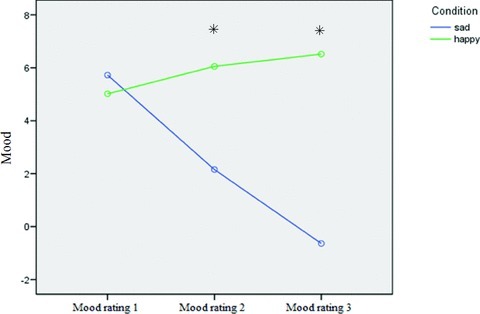
Differences between mood induction groups in self-ratings of mood before and after the two mood inductions. Mean scores for the sad and happy mood groups are shown for baseline mood rating (1), first postmood induction rating (2), and second postmood induction rating (3).

The mean self-ratings for mood on each of the three time points were compared between the sad and happy mood-induced groups by a 2 (Mood type: sad, happy) × 3 (Measurement time point) analysis of variance (ANOVA) (see Fig. 3). Both the main effects of Measurement time point (*F*(1.581, 180.21) = 60.903, *P* < 0.001) and Induced mood type (*F*(1,114) = 54.274, *P* < 0.001) were highly significant. There was also a highly significant interaction effect (*F*(1.581, 180.21) = 180.704, *P* < 0.001). Examination of the means indicated that the change in mean mood self-ratings was much more pronounced for the sad mood group than for the happy mood group. Paired *t*-tests of successive mean mood scores for the sad mood group found that there was a significant decline in its mean mood ratings both between the first and second measurement times (*t*(57) = 7.953, *P* < 0.001) and between the second and third measurement times (*t*(57) = 5.02, *P* < 0.001). The predicted effects of mood induction on the two groups were fully confirmed.

### Internal consistency of stimuli

In the second stage of the study, the focus was directed to determining whether the mood groups differed in their reactions to any of the valenced stimuli, and to whether the stimuli evoked different reaction times for the sample as a whole and differentially for each mood group. The three reaction time measurements taken from the three Stroop trials obtained for each stimulus and for each subject were averaged into one score. These combined scores were considerably more reliable than single observation scores. All of the three-observation composite scores proved to be highly reliable (Cronbach's α statistic ≥ 0.889) for the sample as a whole.

### Group differences on the verbal-emotional Stroop

The difference between the two mood groups in their mean reaction times for each stimulus type in the verbal-emotional Stroop test was tested for significance by conducting a 2 (Group: sad, happy) × 4 (Verbal Valence: sad, fearful, neutral, happy) ANOVA. The results indicate a main effect of mood with sad mood being significantly slower overall in their mean reaction time over the combined set of valences on the verbal-emotional Stroop test, *F*(1, 114) = 4.76, *P*= 0.031, ηp2 = 0.040. Further inspection of the data revealed an interaction effect in mean reaction times between the two mood groups with the sad mood group having significantly longer latencies than the happy mood group for the sad words, *F*(1, 114) = 5.07, *P*= 0.005, ηp2 = 0.043). The other three verbal valences did not differ significantly between groups (all *P* values ≥ 0.05.)

### Group differences on the color Stroop

The difference between the two mood groups in their mean reaction times for the color Stroop test was tested for significance by conducting a one-way ANOVA with mood (sad and happy) as the independent variable and reaction time as the dependent variable. The results of this analysis indicate that there was no significant difference in mean reaction times between the two mood groups on the color Stroop test, *F*(1, 114) = 1.94, *P*= 0.166. Table 2 reports the differences in verbal-emotional Stroop and color Stroop means between the two mood groups.

### Group differences on the facial-emotional Stroop

The difference between the mood groups in their mean reaction times for each stimulus type in the facial Stroop test was tested for significance by conducting a 2 (Group: sad, happy) × 4 (Face Valence: sad, angry, neutral, happy) × 2 (Gender: male, female) ANOVA. First, the findings reveal a main effect of mood with the sad mood group taking significantly longer overall than the happy mood group, *F*(1, 114), = 4.77, *P*= 0.008, ηp2 = 0.040. A significant interaction was found for Mood × Emotional Face *F*(1, 114), = 6.59, *P*= 0.012, ηp2 = 0.048. Comparisons of the mood group means within each of the emotional face types reveal that the mean response times did not differ between the two mood groups for the happy and sad faces, but did differ significantly for the angry-threatening (*t*(114) = 3.818 with adjustment for heterogeneity, *P* < 0.001) and the neutral (*t*(114) = 1.990, *P*= 0.049) emotional faces, with longer latencies for the sad mood group. Also of interest was the impact of facial gender on response time for both groups. Results revealed a significant facial gender by emotion interaction whereby both groups responded slower to neutral female faces compared to male neutral faces *F*(2, 114), = 7.16, *P*= 0.009, ηp2 = 0.059. No other differences concerning facial gender yielded significant differences.

### Group differences on the chairs Stroop

The difference between the two mood groups in their mean reaction times for the chairs Stroop test was tested for significance by conducting a one-way ANOVA with mood as the independent variable and reaction time as the dependent variable. The results of this analysis indicate the difference in mean reaction times between the mood groups was not statistically significant, *F*(1, 114) = 2.86, *P*= 0.093. Table 3 reports the differences in facial-emotional means and chairs means between the two mood groups.

## Discussion

Despite the many efforts to investigate attentional interference in sad mood, the specific valenced stimuli that cause interference have not been unequivocally established. Compared to depression research utilizing the emotional Stroop (e.g., Lim and Kim 2005), less work has been done to investigate emotional Stroop performance as a function of mood in nondepressed participants put into a sad mood, and results have been mixed (Chepenik et al. 2007). The aim of the present study was to learn whether attentional interference occurred for subjects in sad mood states for emotionally relevant stimuli (mood-congruence), and to determine whether this interference occurred for both valenced words and valenced faces. We were unable to locate any prior studies that have evaluated valence and modality-specific attentional interferences using three versions of the Stroop task across the same sample of participants. The current design also included reaction times for naming colored chairs as a control condition. Nonsignificant results for this control measure reinforce the inference that group differences found in this study is likely due to the emotion aspect of the stimuli. Equally relevant to this point is the nonsignificant group difference on the color Stroop task administered for an assessment of basic processing speed and flexibility.

### Verbal-emotional findings

Given the frequent co-occurrence of anxiety and sad mood (Mineka et al. 1998), it is necessary to include both anxiety-laden and depression-laden content to better differentiate their relative contribution to verbal Stroop interference, which was done in the present study. The present finding that depressive words lead to significant mood group differences on the verbal-emotional Stroop task replicates both long-standing research (e.g., Hill and Knowles 1991; Mitterschiffthaler et al. 2008) and the most current work on this topic (Koster et al. 2010). Sad mood participants had longer reaction times for sad words on the verbal-emotional Stroop and interestingly, these depressive words consisted of self-describing adjectives such as “worthless.” However, several authors have assessed attention to emotional words in sad and depressed patients and have failed to find attentional interference with reactions to negative stimuli. For instance, Macleod et al. (1996) concluded their depressed sample did not show evidence of a bias for negative verbal stimuli. One such possibility for this could be the use of a heterogeneous sample of participants who were not matched in age, and which consisted of both older inpatient and younger outpatient participants.

The present verbal-emotional Stroop results both replicate and extend the findings of Gotlib and McCann's (1984) that dysphoric students take significantly longer to name the color of words having depressed content than words having anxiety content, although the present study obtained the same finding for subjects in a transitory induced mood state. It is noteworthy to mention that Gotlib's study did not directly assess anxiety through the use of threatening words and thus the depressive Stroop effect found could possibly be a reflection of anxiety rather than dysphoric mood.

### Facial-emotional Stroop findings

A principal aspect of the present study was to investigate how people in a sad mood attend to emotional faces compared to those in a happy mood. The pertinent finding of this study is that, contrary to previous claims (e.g., Williams et al. 1997); people in a sad mood do show an attentional bias. Specifically, it was found that participants in the sad mood condition took significantly longer to attend to angry-threatening facial expressions compared to those in the happy mood condition. Contrary to what was predicted, the present results did not support cross-modality for mood-congruent stimuli. Specifically, mood-congruent sad faces in the visual domain did not lead to a difference between mood groups in attentional interference whereas mood-congruent depressive words in the verbal domain did lead to such a difference, with sad participants taking longer. Sad mood participants also took longer in the neutral face condition, replicating the work of Bouhuys et al. (1995) that neutral faces are not necessarily viewed as valence-free by sad mood participants. The current study also took into consideration the impact of the facial gender of visual stimuli on attentional interference in the different mood groups. Interestingly, gender impacted both sad and happy mood groups, with both groups having longer reaction times to neutral female faces compared to neutral male faces.

The present results do not agree with those of Williams et al. (1996), who found that an attentional bias toward threatening stimuli was associated with anxiety but not depression, which might be considered to represent a sad mood condition. The question arises of how differences between the Stroop findings obtained by Williams et al. (1996) and those of the present investigation are to be explained. One possibility might be the divergence in results may be explained in terms of the variation in stimuli. In other words, verbal stimuli used in previous work may not have been potent enough to elicit the attentional interference that threatening (angry) faces clearly did in the present study. A threatening word is symbolic of danger whereas an angry face may be more personally salient. The present study's results also do not fully align with studies that found a mood-congruent bias for sad faces in clinical samples (e.g., Gotlib et al. 2004). One possible explanation for this difference in findings is that sad faces may merely signal another's emotional state and may not have direct relevance to the sad person, whereas an angry face is a direct signal of personal disapproval and dislike and may be more likely to be relevant to a sad person. Interestingly, Schmid et al. (2011) suggest that differences between the two mood states arise primarily from a difference in face processing strategy. Specifically, in their eye-tracking experiment, Schmid et al. found that sad mood participants applied a featural face processing strategy zooming into emotion-relevant areas (eyes, mouth) whereas happy mood participants processed faces using a configural face processing strategy (spatial and structural encoding of faces).

The results of the present study provide additional support for the idea that participants in a sad mood are sensitive to angry faces and maintain their attention on these threatening faces (Leyman et al. 2006). Indeed these findings are in concert with Leyman et al.; however, as noted by the authors themselves, the comparison of only neutral and angry faces is limited. It is possible that the single use of angry versus neutral faces is sensitive to both arousal and valence. This study has improved upon the design of the Leyman et al. (2006) study by including a wider range of emotions. It offers additional support that threatening angry faces do indeed capture the attention of those in a sad mood. We can thus extrapolate from this that vigilance for threatening faces is not an exclusive function of anxiety as previously reported (see review paper by Mogg and Bradley 2005). Once again, a possible explanation for why the present study found significance for threat stimuli in sad mood while others have found this primarily in anxious samples (e.g.,Van Honk et al. 2001; Mogg and Bradley 2002; Mogg et al. 2007) can perhaps be due to the exclusive use of verbal stimuli. Valenced verbal stimuli, although highly valuable for the study of attentional bias, may lack the potency necessary to elicit an externally driven attentional bias, namely for threatening angry faces. For instance, a survey of the referenced articles in the review paper by Mogg and Bradley (2005) reveals that with the exception of one study that utilized emotional face stimuli (Bradley et al. 1999), all other experiments utilized emotional words. If the suggestion about faces having more strength for threat detection holds true, this could partially explain the lack of findings for an external threat bias in both sad mood and depressed samples. Second, although highly speculative at this point, it is not entirely convincing that threatening faces are strictly signals of danger in the environment and thus belong exclusively in the anxiety attentional bias camp. An angry face can possibly be a signal of impending doom and aggression for the anxious observer or a signal of disapproval and rejection for the sad or depressed observer. Lastly, contrary to our hypothesis, happy mood participants did not pay more attention to positive stimuli. In the present study, these participants paid less attention to negative stimuli suggesting that perhaps the *protective bias* can also be defined by what healthy controls do not attend to, namely negative stimuli.

## Summary and Conclusions

The present study investigated attentional interference for both emotional words and emotional faces across a wide range of valences. Overall, the present results support earlier studies indicating that people in a sad mood show slower reaction times to processing affective. A limitation of our study merits comment. To assess self-processing within an emotional context, it has been recommended that valenced words be restricted to self-referencial stimuli (Fossati et al. 2003). The present study controlled for many aspects of the verbal stimuli (e.g., arousal, word length) but was not exclusively categorized by self-referential words. Overall, the present results support earlier studies indicating that people in a sad mood show slower reaction times to processing affective information (Leppanen 2006), particularly when the stimuli are negatively valenced (Baumeister et al. 2001). We have identified specific verbal and facial emotional cues that lead to interference in attention for those in a sad mood. The clearest finding of our experiment is that attentional interference for mood-congruent depressive words and threat-related angry facial expressions have significant influences on attentional processes among people in a sad mood state. Furthermore, emotionally ambiguous stimuli such as neutral faces were attended to longer by sad mood participants suggesting that perhaps these participants did not see the neutral faces as valence-free, which converges with the work of Leppanen et al. (2004), who reported a biasing of neutral faces in depressed patients and Bouhuys et al. in a sad-induced sample. Support for this finding can be found in the neuroimaging literature which points to elevated physiological activity of the amygdala for emotionally neutral stimuli (e.g., neutral faces) among sad or depressed subjects, possibly resulting in such subjects interpreting these stimuli as having emotional significance (Drevets 2001). On the basis of these findings, we suggest that theoretical frameworks regarding altered cognitive processes in sad mood states need to accommodate attentional interference for both valenced and unvalenced words and faces.
